# Epac1 Restores Normal Insulin Signaling through a Reduction in Inflammatory Cytokines

**DOI:** 10.1155/2018/3809092

**Published:** 2018-07-19

**Authors:** Elizabeth Curtiss, Youde Jiang, Li Liu, Claire Hawthorne, Jessica Zhang, Jena J. Steinle

**Affiliations:** ^1^Department of Anatomy and Cell Biology, Wayne State University School of Medicine, Detroit, MI, USA; ^2^Department of Ophthalmology, Wayne State University School of Medicine, Detroit, MI, USA

## Abstract

We have previously reported that Epac1 reduced inflammatory cytokines, which is protective to the diabetic retina. We have also published that impaired insulin signaling occurs in the diabetic retina. A reduction in interleukin-1 beta (IL-1*β*) and tumor necrosis factor alpha (TNF*α*) by Epac1 could potentially restore normal insulin signal transduction. Confocal microscopy was performed to localize the insulin receptor in the retina of Epac1 floxed and endothelial cell-specific Epac1 knockout mice. Whole retinal lysates from Epac1 floxed and endothelial cell-specific Epac1 knockout mice were used to investigate proteins involved in the insulin signaling cascade. Primary human REC were cultured in normal and high glucose followed by Epac1 agonist treatment or transfection with IL-1*β* or TNF*α* siRNA for protein analyses of insulin signaling proteins. Decreased expression of the insulin receptor was observed in the Epac1 knockout mouse retinal vasculature compared to floxed littermates. Work in mice showed that loss of Epac1 decreased insulin signaling proteins. Treatment with an Epac1 agonist decreased p38 and JNK signaling and increased insulin signaling, as did inhibition of IL-1*β* or TNF*α* using siRNA when added to REC grown in high glucose. Taken together, Epac1 can restore normal insulin signaling in the retinal vasculature through reductions in inflammatory cytokines.

## 1. Introduction

While diabetic retinopathy is the leading cause of blindness in working-age adults, there are currently limited treatment options for early phases of the disease, despite decades of research. Inflammatory cytokines have been shown to be one of the initiating factors in damage to the diabetic retina [1, 2]. Exchange protein for cAMP (Epac1) has been shown to be protective against inflammatory cytokines in vascular endothelial cells [3]. In the vasculature, Epac1 downregulates interleukin 6- (IL-6-) mediated inflammatory processes [4], activates integrins involved in the adhesion of endothelial cells to the basement membrane to limit vascular permeability [5], and maintains the endothelial barrier [6]. Epac1 mediates the cAMP-dependent activation of Rap1 [7]. In addition to blood vessels, Epac1 has been found to be expressed in the kidney, adipose, central nervous system, ovary, and uterus [8]. Furthermore, Epac1 has been localized to the retina [9] and is expressed on human retinal endothelial cells (REC) [10]. Research has shown that inhibiting inflammatory mediators is protective to the retina. A previous work in our lab has shown that Epac1 blocks the proinflammatory mediator tumor necrosis factor alpha (TNF*α*) and interleukin-1 beta (IL-1*β*) in REC and endothelial cell-specific conditional knockout mice [10].

In addition to inflammation having a prominent role in diabetic retinal pathology, impaired insulin signaling occurs in the diabetic retina, particularly in type 2 diabetes [11, 12]. These two pathways are potentially interconnected, as TNF*α* has been shown to be a key player in disrupting insulin signaling [12, 13]. TNF*α* has been found in the vascular walls of proliferative diabetic retinopathy [14]. Work has shown that TNF*α* mediates apoptosis early in the development of diabetic retinopathy and is a major contributor to long-term histopathological alterations [1]. We have previously reported TNF*α* increased insulin receptor substrate 1 (IRS-1)^Ser307^ phosphorylation in REC, thus blocking insulin signal transduction [12]. In addition to TNF*α*, the proinflammatory cytokine, IL-1*β*, has been implicated in diabetes [15]. Gevokizumab (a monoclonal IgG2 antibody against IL-1*β*) improved pancreatic *β*-islet cell function and glycemia, as well as reduced the load of systemic inflammatory mediators in patients [16]. IL-1*β* expression is increased in the retina of diabetic rodents [17] and plays a critical role in neuroinflammation and diabetic retinal pathology [18]. Elevated levels of IL-1*β* increase diabetes susceptibility through increased insulin resistance [18].

In this study, we used Epac1 endothelial cell-specific knockout mice and REC in culture to investigate whether Epac1 restores normal insulin signaling through its inhibition of TNF*α* and IL-1*β*.

## 2. Materials and Methods

### 2.1. Mice

Experiments were done using Epac1 endothelial cell-specific knockout mice [10]. Mice of both sexes at 3 months of age were used. Animal procedures were reviewed and approved by the Institute Animal Care and Use Committees of Wayne State University School of Medicine (17-07-301) and conform to NIH guidelines.

### 2.2. Immunohistochemistry for the Insulin Receptor

Four Epac1 floxed and Epac1 Cdh5 Cre-lox mice were euthanized with carbon dioxide and cervical dislocation. The eyes were removed and placed in 4% paraformaldehyde in PBS (1X; 137 mM NaCl, 2.7 mM KCl, 10 mM Na2HPO4, and 1.8 mM KH2PO4, pH 7.4) overnight. The next day, the eyes were transferred to 0.1 M PBS containing 30% sucrose for cryoprotection. Using a cryostat, ten-micron sections were cut and stored at 80°C until use. The sections were rinsed in PBS and put in 5% normal goat serum for 1 h at room temperature to block nonspecific staining, followed by incubation with isolectin GS-IB4 to label the retinal vasculature (Alexa Fluor 488 conjugate, 1 : 100, Life Technologies) [10] and anti-insulin receptor (1 : 500, Abcam) at 4°C overnight. After incubation, the sections were rinsed in 0.3% Triton/PBS followed by incubation with secondary antibody goat anti-rabbit conjugated to Alexa Fluor 594 (1 : 1000, Life Technologies) for 2 h at room temperature. Slides were rinsed in PBS and counterstained with 4′,6-diamidino-2-phenylindole (DAPI). The slides were examined with a Leica confocal microscope (Buffalo Grove, IL).

### 2.3. Retinal Endothelial Cell Culture (REC)

Primary REC were purchased from Cell Systems Corporation (CSC, Kirkland, WA). Cells were grown in normal (5 mM) or high-glucose (25 mM) Cell Systems Medium. The media were supplemented with microvascular growth supplement (Invitrogen), 10 *μ*g/mL gentamycin, and 0.25 *μ*g/mL amphotericin B. For experiments, the cells were exposed to high-glucose medium for a minimum of 3 days. Only the cells prior to passage 5 were used.

### 2.4. Cell Treatments

REC grown in normal and high glucose were quiesced by incubating in normal or high-glucose medium without MVGS for 24 hours prior to treatment with 8-CPT-2′-O-Me-cAMP (an Epac1 agonist) at 10 *μ*M for 2 h. Additional REC in normal and high glucose were transfected with either IL-1*β* siRNA or TNF*α* siRNA (Dharmacon, Lafayette, CO), or scrambled siRNA at a final concentration of 20 nM using GenMute (SignaGen Laboratories, Rockville, MD), according to the manufacturer's instructions. Twenty-four hours after transfection, the cells were processed for protein analyses.

### 2.5. Western Blotting

Whole retinal lysates or REC were collected into lysis buffer containing protease and phosphatase inhibitors. Equal amounts of protein were loaded and separated onto precast Tris-glycine gels (Invitrogen, Carlsbad, CA) and then blotted onto a nitrocellulose membrane. The membranes were blocked in TBST (10 mM Tris-HCl buffer, pH 8.0; 150 mM NaCl, and 0.1% Tween 20) and 5% BSA and then incubated with corresponding primary antibodies, followed by incubation with secondary antibodies labeled with horseradish peroxidase. Primary antibodies used included Epac1, IL-1*β*, Rap 1 (Abcam, Cambridge, MA), phosphorylated p38 MAPK, total p38 MAPK, phosphorylated JNK, total JNK, phosphorylated insulin receptor (Tyr 1150/1151), total insulin receptor, phosphorylated Akt (Ser473), total Akt, Akt2, phosphorylated IRS-1 (Ser307), total IRS-1 (Cell Signaling Corp, Danvers, MA), and beta-actin (Santa Cruz, CA). Phosphorylated proteins were compared to total protein levels, and the other proteins were compared to beta-actin. Antigen-antibody complexes were identified using a chemiluminescence reagent kit (Thermo Scientific, Pittsburgh, PA). Imaging was performed using a C500 (Azure Biosystems, Dublin, CA).

### 2.6. ELISA

A cleaved caspase 3 ELISA (Cell Signaling Corp, Danvers, MA) was done according to the manufacturer's instructions on both mouse whole retinal lysates and REC. A TNF*α* ELISA (Fisher Scientific, Pittsburgh, PA) was performed based upon the manufacturer's instructions with the following exceptions: 100 *μ*g protein of each sample was added into wells, and the incubation of samples and primary antibody occurred for 24 h at 4°C.

### 2.7. Statistical Analyses

Prism software 6.0 (GraphPad, La Jolla, CA) was used for statistical analyses. A nonparametric Kruskal-Wallis with Dunn's post hoc test was used for cell culture data. A one-way ANOVA with Student-Newman-Keul's post hoc test was used for cell culture work. *P* < 0.05 was considered to be significant.

## 3. Results

### 3.1. Insulin Receptor Colocalized with Isolectin B4 in the Retina Epac1 Floxed Mice

**Figure 1:**
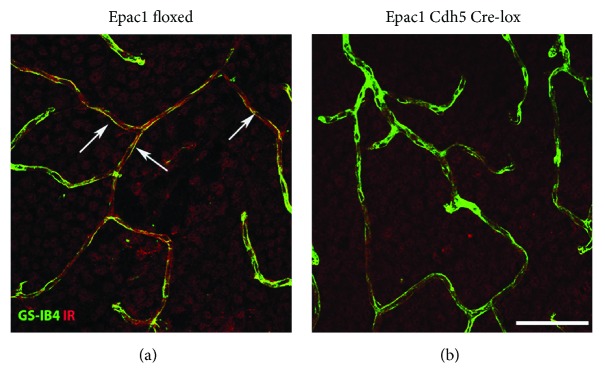
Insulin receptor (red) and isolectin B4 (green) colocalize in the retina of the Epac1 floxed mouse (a) with little staining for insulin receptor in the vasculature of the Epac1 Cre-lox mice (b). *N* = 5. Scale bar = 50*μ*m.

We wanted to colocalize the insulin receptor within the retinal vasculature of the Epac1 mice.  Figure 1 shows that insulin receptor and isolectin B4 colocalize in the retina of the Epac1 floxed mice with little minimal staining for the insulin receptor in the vasculature of the Epac1 Cre-lox mice.

### 3.2. Loss of Epac1 in Mouse Retinal Vasculature Leads to Decreased Insulin Signaling

**Figure 2:**
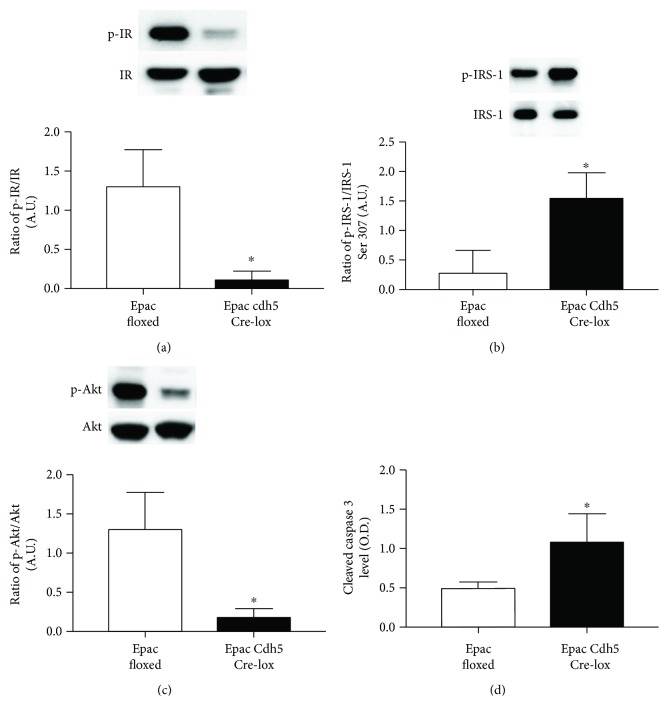
Western blotting for the ratio of phosphorylated insulin receptor on tyrosine 1150/1151 (p-IR) (a), IRS-1^Ser307^ (p-IRS-1) (b), and Akt (p-Akt) (c) to total protein in whole retinal lysates from Epac1 floxed mice or Epac1 Cdh5 Cre-lox mice. ELISA results for cleaved caspase 3 (d). ^∗^*P* < 0.05 versus Epac1 floxed mice. *N* = 5 for all mice. Data are mean ± SEM.

To determine the effect of Epac1 on insulin signaling, we analyzed the effect of the loss of Epac1 in mouse whole retinal lysates. Phosphorylated insulin receptor (IR) and phosphorylated Akt were significantly reduced in the Epac1 Cre-lox mice compared to their floxed littermates (Figures 2(a) and 2(c)). Phosphorylated IRS-1^Ser307^ and cleaved caspase 3 levels were significantly increased with loss of Epac1 in the mouse retinas (Figures 2(b) and 2(d)), suggesting that the loss of Epac1 in the retinal vasculature of mice disrupts normal insulin signaling, leading to increased apoptosis.

### 3.3. Epac1 Agonist Treatment Led to Increased Rap1 and Decreased p38 MAPK and JNK

**Figure 3:**
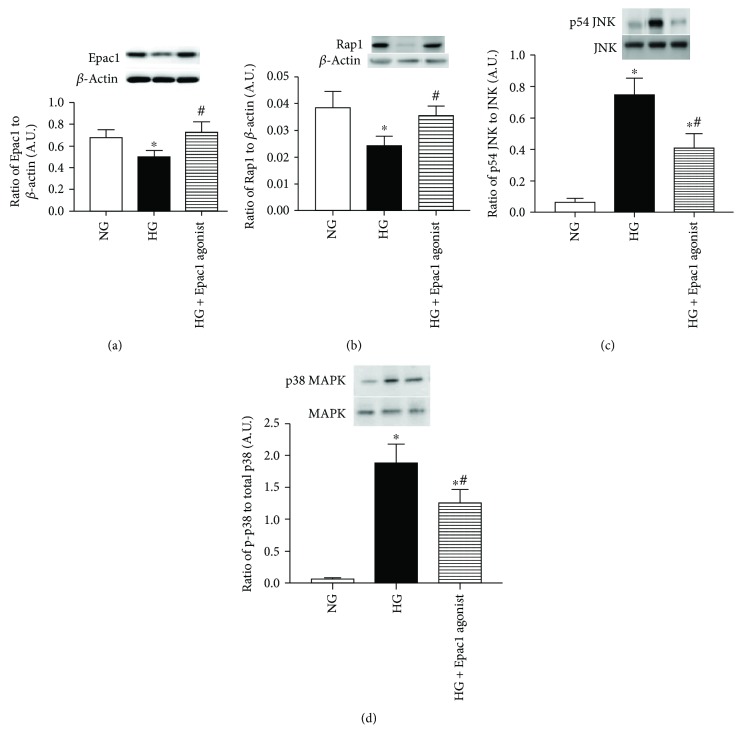
(a) Western blotting for the ratio of Epac1 to *β*-actin in REC grown in normal glucose (NG), high glucose (HG), and HG + 10 *μ*M 8-CPT-2′-O-Me-cAMP (Epac1 agonist). (b) Western blotting for the ratio of Rap 1 to *β*-actin. (c, d) Western blotting for the ratio of phosphorylated p38 MAPK and JNK to total P38 or JNK protein. ^∗^*P* < 0.05 versus NG, ^#^*P* < 0.05 versus HG. *N* = 3 or 4 for all groups. Data are mean ± SEM.

To determine how Epac1 may regulate insulin signaling cascade *in vitro*, we treated REC grown in high glucose with an Epac1 agonist and compared protein levels to REC grown in normal and high glucose. We first confirmed that Epac1 significantly increased Epac1 levels (Figure 3(a)). Compared to the high glucose-only group, the Epac1 agonist increased the levels of Rap1, while decreasing the levels of phosphorylated p38 MAPK and JNK (Figures 3(b)–3(d)).

### 3.4. Epac1 Agonist Treatment Restores Normal Insulin Signaling

**Figure 4:**
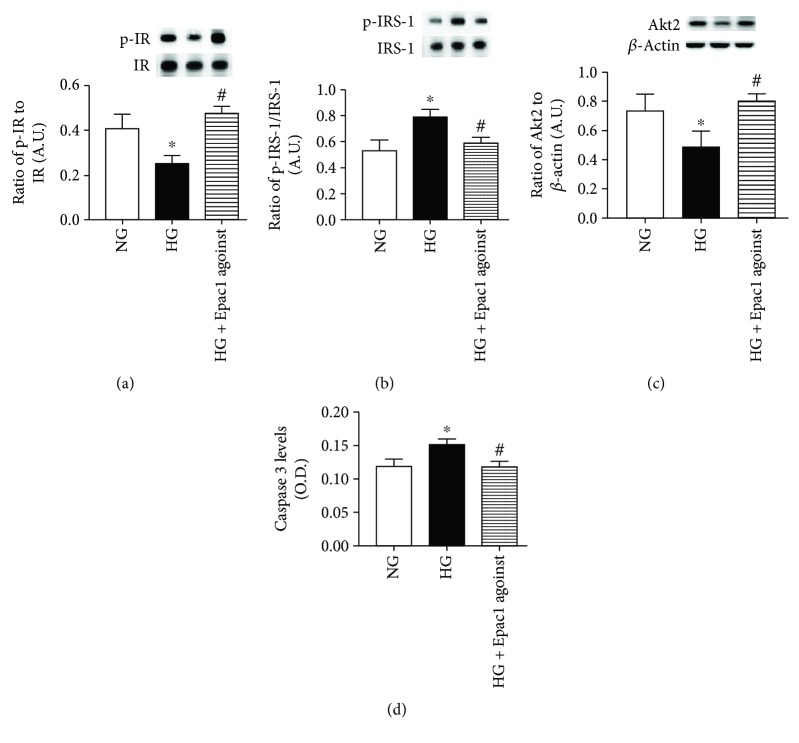
(a, b) Western blotting for the ratio of phosphorylated insulin receptor on tyrosine 1150/1151 (p-IR) and IRS-1^Ser307^ (p-IRS-1) to total protein. (c) Western blotting for the ratio of Akt2 to *β*-actin. (d) ELISA results for cleaved caspase 3 levels. ^∗^*P* < 0.05 versus NG, ^#^*P* < 0.05 versus HG. *N* = 4 or 5 for all groups. Data are mean ± SEM.

Compared to the high glucose-only group, the Epac1 agonist increased the levels of phosphorylated insulin receptor and Akt2, while decreasing the levels of phosphorylated IRS-1^Ser307^ and cleaved caspase 3 (Figures 4(a)–4(d)). Taken together, the data indicates that Epac1 restores normal insulin signaling in REC grown in high glucose.

### 3.5. Transfection with IL-1*β* siRNA Decreases IL-1*β* Levels and Increases Insulin Signaling in High Glucose

**Figure 5:**
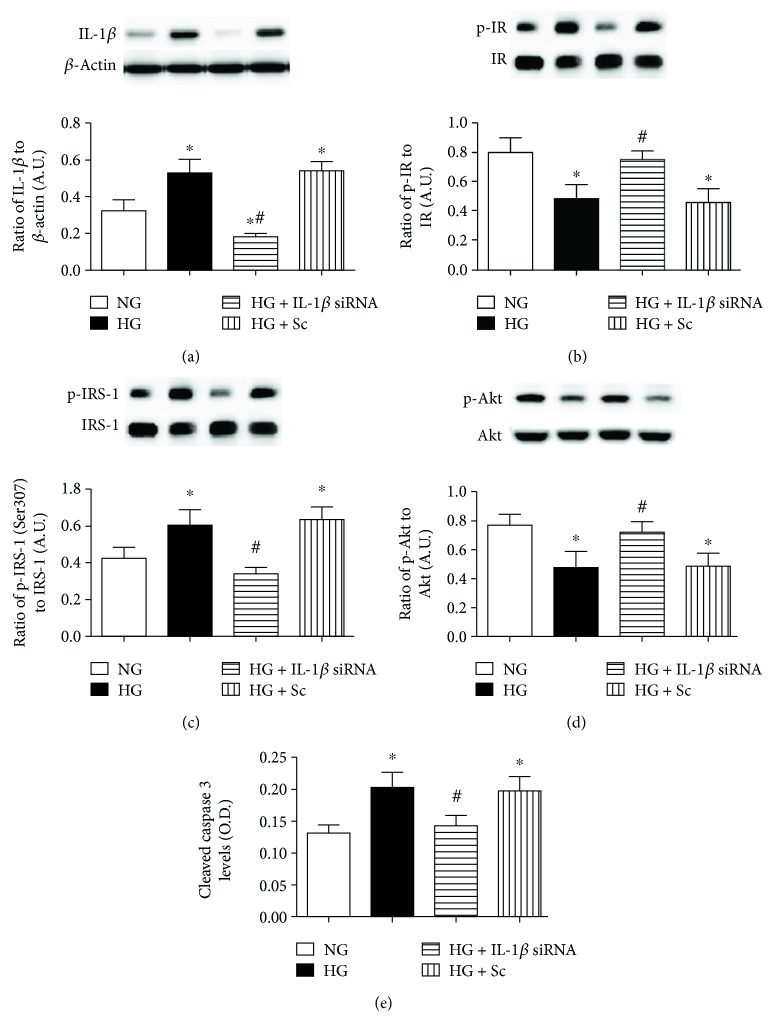
(a) Western blotting for the ratio of IL-1*β* siRNA to *β*-actin in REC grown in normal glucose (NG) and high glucose (HG). Some REC grown in HG were transfected with IL-1*β* siRNA or scrambled siRNA (sc). (b–d) Western blotting for the ratio of phosphorylated insulin receptor on tyrosine 1150/1151 (p-IR), IRS-1^Ser307^ (p-IRS-1), and Akt (p-Akt) to total protein. (e) ELISA results for cleaved caspase 3 levels. ^∗^*P* < 0.05 versus NG, ^#^*P* < 0.05 versus HG. *N* = 4 or 5 for all groups. Data are mean ± SEM.

We have previously reported Epac1 decreases IL-1*β* expression in REC in high glucose [10]. To determine if insulin signaling can be restored by a reduction in the inflammatory cytokines regulated by Epac1, we treated REC grown in high glucose with IL-1*β* siRNA or scrambled siRNA. We first confirmed successful knockdown of IL-1*β* with IL-1*β* siRNA transfection (Figure 5(a)). IL-1*β* siRNA transfection led to a significant increase in phosphorylation of the insulin receptor and Akt compared to REC grown in high glucose only (Figures 5(b) and 5(d)). In addition, IL-1*β* siRNA transfection led to a significant reduction in phosphorylated IRS-1^Ser307^ and cleaved caspase 3 levels compared to REC cultured only in high glucose (Figures 5(c) and 5(e)). There was no significant difference in any protein levels in the REC transfected with IL-1*β* siRNA compared to cells in normal glucose, suggesting a restoration of insulin signaling when IL-1*β* is blocked.

### 3.6. Transfection with TNF*α* siRNA Decreases TNF*α* Levels, Phosphorylated IRS-1^Ser307^ Levels, and Cleaved Caspase 3 Levels, as well as Increases Phosphorylated Insulin Receptor and Phosphorylated Akt Levels

**Figure 6:**
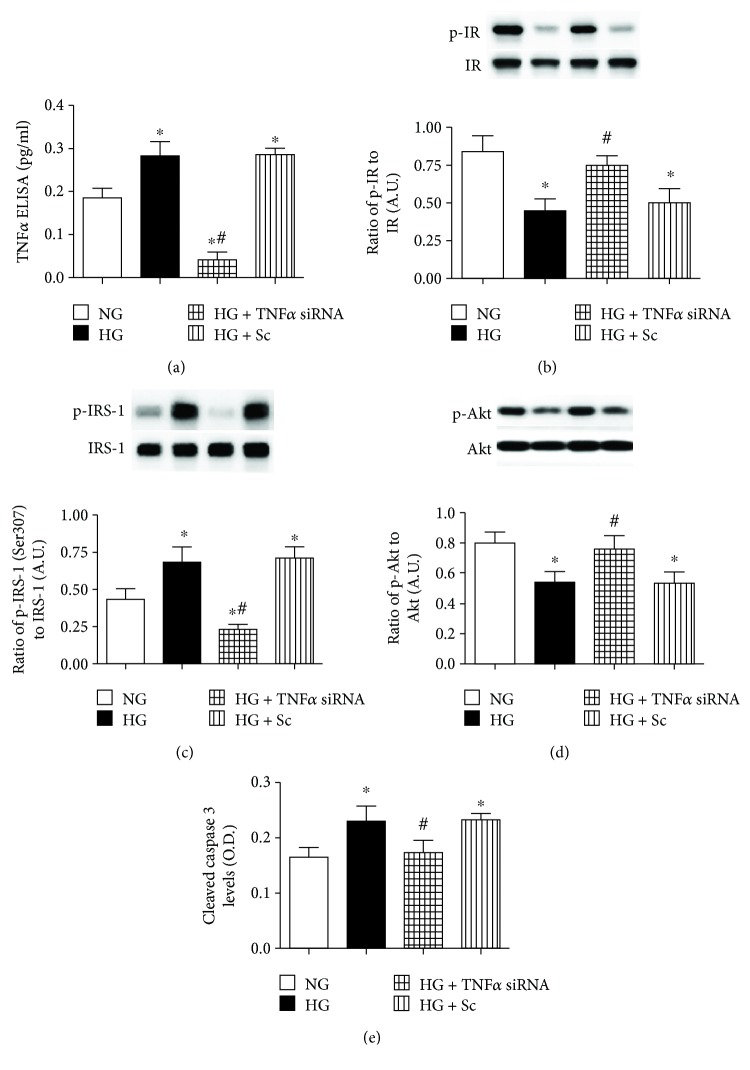
(a) Western blotting for the ratio of TNF*α* siRNA to *β*-actin in REC grown in normal glucose (NG) and high glucose (HG). Some REC grown in HG were transfected with TNF*α* siRNA or scrambled siRNA (sc). (b–d) Western blotting for the ratio of phosphorylated insulin receptor on tyrosine 1150/1151 (p-IR), IRS-1^Ser307^ (p-IRS-1), and Akt (p-Akt) to total protein. (e) ELISA results for cleaved caspase 3 levels. ^∗^*P* < 0.05 versus NG, #*P* < 0.05 versus HG. *N* = 4 or 5 for all groups. Data are mean ± SEM.

**Figure 7:**
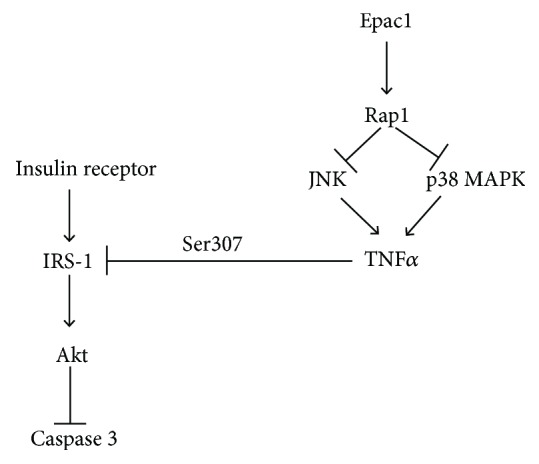
Schematic of the proposed role of Epac1 in the insulin signaling cascade.

We have previously reported Epac1 decreases TNF*α* levels in REC in high glucose [10]. To determine if a reduction of TNF*α* could increase insulin signaling in high glucose, we treated REC grown in high glucose with TNF*α* siRNA or scrambled siRNA. We confirmed successful knockdown of TNF*α* using TNF*α* siRNA transfection (Figure 6(a)). Data indicates that the inhibition of TNF*α* significantly increased insulin receptor and Akt phosphorylation (Figures 6(b) and 6(d)). Correspondingly, TNF*α* siRNA transfection significantly decreased phosphorylated IRS-1^Ser307^ and cleaved caspase 3 levels compared to REC in high glucose only (Figures 6(c) and 6(e)). Taken together, the data suggests that the inhibition of TNF*α* can restore normal insulin transduction in REC grown in high glucose.  Figure 7 is a schematic of a potential mechanism by which Epac1 can decrease TNF*α* to block IRS-1 phosphorylation on serine 307 to maintain normal insulin signal transduction.

## 4. Discussion

Disruption of the normal insulin signaling cascade occurs in type 1 and type 2 diabetes. Prior work in our laboratory has found that *β*-adrenergic receptors restored normal insulin signaling in *β*2-adrenergic receptor-deficient mice [19]. We also found that *β*-adrenergic receptor agonists can decrease inflammatory cytokines, such as TNF*α*, which disrupt insulin signaling [12]. Additionally, we have previously reported that an Epac1 agonist decreases TNF*α* and IL-1*β* levels in the retina [10]. Thus, we questioned if using Epac1 could lead to a restoration of normal insulin signaling through a reduction in inflammatory mediators.

Using confocal microscopy, we found that the insulin receptor is in the retinal vasculature of mice. This was further confirmed through Western blotting and ELISA analyses of insulin signaling proteins in Epac1 endothelial cell-specific knockout and Epac1 floxed mice. We found that loss of Epac1 in the mouse retinal vasculature led to decreased insulin signaling, based upon reduced phosphorylation of insulin receptor and Akt2 levels. Akt2 is a key to insulin function, as mice lacking Akt2 have insulin resistance and a diabetes mellitus-like syndrome occurred [20]. In addition, Akt2 has been shown to regulate glucose transport in cultured adipocytes [21]. We also found that Epac1 knockout mice had increased levels of phosphorylated IRS-1^Ser307^ and cleaved caspase 3 in whole retinal lysates. IRS-1 is a major substrate of the insulin receptor, which activates PI3K in response to insulin, leading to Akt phosphorylation and the eventual uptake of glucose [22].

To further understand the role of Epac1 in regulating insulin signaling through actions on inflammatory mediators, we cultured human REC in normal and high glucose. We found that Epac1 increased Rap1 verifying Epac1's role in activating Rap1 [23]. Epac1 led to decreased levels of phosphorylated p38 MAPK. In other work, cAMP inhibited the activation of p38, leading to suppression of NF-*κβ* in fibroblasts [24]. Further, Epac1 agonist treatment also decreased the levels of phosphorylated JNK. JNK has been shown to be required for TNF*α*-mediated apoptosis [25]. cAMP has been shown to significantly inhibit TNF*α*-induced JNK activation [26].

Epac1 agonist treatment also decreased the IL-1*β* levels in REC, confirming our previous results [10]. In a study by Gao et al., they reported that IL-1*β* inhibited insulin signaling through decreased expression of key proteins in the insulin signaling cascade including IRS-1 and GLUT4 in human adipocytes [27]. Similarly, IL-1*β* has been shown to inhibit IRS-1 expression, leading to decreased insulin-induced glucose transport in adipocytes [28]. IL-1*β* also induced insulin resistance in keratinocytes through the p38 MAPK pathway [29]. Furthermore, in a study using 3T3-L1 mouse adipocytes and human adipocytes, IL-1*β* inhibited the insulin-induced phosphorylation of the insulin receptor *β* subunit, IRS-1, and Akt, as well as suppressed insulin-induced glucose transport and lipogenesis [30]. In the current study, when we transfected REC grown in high glucose with IL-1*β* siRNA, we found that IL-1*β* inhibition led to increased insulin receptor actions. There were decreased levels of phosphorylated IRS-1^Ser307^ and cleaved caspase 3, confirming that IL-1*β* has a role in regulating the insulin signaling cascade.

In addition to the inflammatory mediator IL-1*β*, we have shown that Epac1 can reduce TNF*α* [10]. Studies have indicated that TNF*α* disrupts insulin signaling through phosphorylation of IRS-1 on serine 307 [31, 32]. Additionally, the work has shown TNF*α* can increase suppression of cytokine signaling 3 (SOCS3) levels [33]. The increase in SOCS3 levels blocks normal insulin receptor phosphorylation on tyrosine 960, leading to REC apoptosis [12]. Our findings support the previous work, loss of TNF*α* increased levels of phosphorylation of the insulin receptor and Akt, with decreased levels of phosphorylated IRS-1^Ser307^ and cleaved caspase 3.

## 5. Conclusions

Taken together, our data suggest Epac1 restores insulin signaling through a reduction in inflammatory cytokines. Using conditional knockout mice for Epac1 in endothelial cells, we found Epac1 increases insulin signaling. This data was confirmed in REC, as treatment with an Epac1 agonist increased insulin receptor phosphorylation and Akt2 levels. In addition, the inhibition of IL-1*β* and TNF*α* in REC grown in high glucose decreased phosphorylated IRS-1^Ser307^ and cleaved caspase 3 levels, while increasing phosphorylation of the insulin receptor and Akt. Taken together, the data suggest that while high glucose can lead to increased inflammatory mediators and depressed insulin signal transduction, Epac1 can reduce these inflammatory mediators to restore normal insulin signaling.

## Data Availability

The data used to support the findings of this study are available from the corresponding author upon request.
