# Erratum to “Epac1 Restores Normal Insulin Signaling through a Reduction in Inflammatory Cytokines”

**DOI:** 10.1155/2019/4524179

**Published:** 2019-06-18

**Authors:** Elizabeth Curtiss, Youde Jiang, Li Liu, Claire Hawthorne, Jessica Zhang, Jena J. Steinle

**Affiliations:** ^1^Department of Anatomy and Cell Biology, Wayne State University School of Medicine, Detroit, MI, USA; ^2^Department of Ophthalmology, Wayne State University School of Medicine, Detroit, MI, USA

In the article titled “Epac1 Restores Normal Insulin Signaling through a Reduction in Inflammatory Cytokines” [1], it was found that the western blots in Figures 5(b) and 5(c) were duplicated. This error happened during the production process. The corrected figure is shown below.

## Figures and Tables

**Figure 1:**
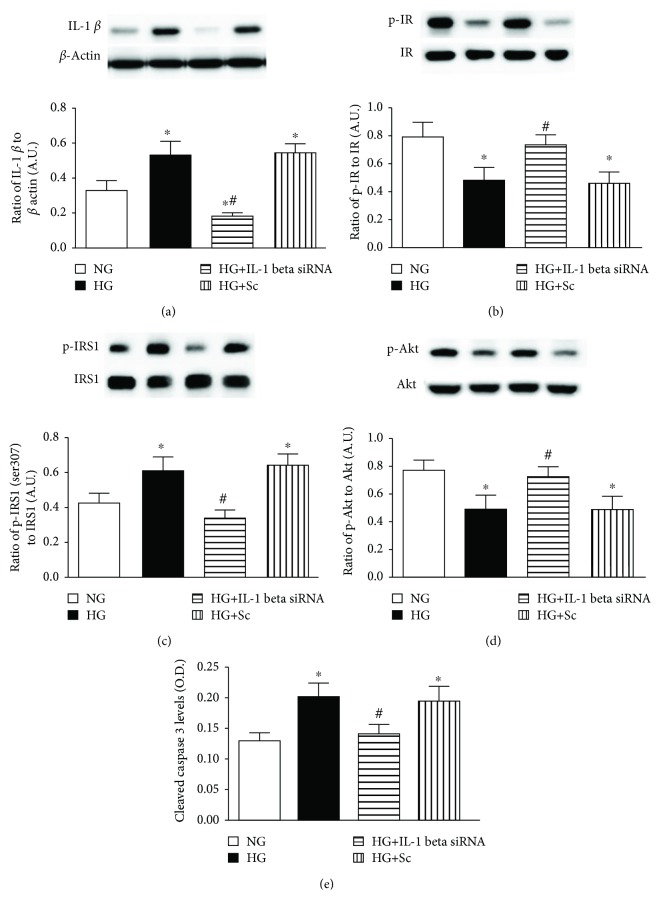
(a) Western blotting for the ratio of IL-1*β* siRNA to *β*-actin in REC grown in normal glucose (NG) and high glucose (HG). Some REC grown in HG were transfected with IL-1*β* siRNA or scrambled siRNA (sc). (b–d) Western blotting for the ratio of phosphorylated insulin receptor on tyrosine 1150/1151 (p-IR), IRS-1Ser307 (p-IRS-1), and Akt (p-Akt) to total protein. (e) ELISA results for cleaved caspase 3 levels. ^∗^*P* < 0.05 versus NG, ^#^*P* < 0.05 versus HG. *N* = 4 or 5 for all groups. Data are mean ± SEM.
